# PhycoCosm, a comparative algal genomics resource

**DOI:** 10.1093/nar/gkaa898

**Published:** 2020-10-26

**Authors:** Igor V Grigoriev, Richard D Hayes, Sara Calhoun, Bishoy Kamel, Alice Wang, Steven Ahrendt, Sergey Dusheyko, Roman Nikitin, Stephen J Mondo, Asaf Salamov, Igor Shabalov, Alan Kuo

**Affiliations:** US Department of Energy Joint Genome Institute, Lawrence Berkeley National Laboratory, Berkeley, CA 94720, USA; Department of Plant and Microbial Biology, University of California Berkeley, Berkeley, CA 94720, USA; Environmental Genomics and Systems Biology, Lawrence Berkeley National Laboratory, Berkeley, CA 94720, USA; US Department of Energy Joint Genome Institute, Lawrence Berkeley National Laboratory, Berkeley, CA 94720, USA; US Department of Energy Joint Genome Institute, Lawrence Berkeley National Laboratory, Berkeley, CA 94720, USA; Environmental Genomics and Systems Biology, Lawrence Berkeley National Laboratory, Berkeley, CA 94720, USA; US Department of Energy Joint Genome Institute, Lawrence Berkeley National Laboratory, Berkeley, CA 94720, USA; Environmental Genomics and Systems Biology, Lawrence Berkeley National Laboratory, Berkeley, CA 94720, USA; US Department of Energy Joint Genome Institute, Lawrence Berkeley National Laboratory, Berkeley, CA 94720, USA; Department of Plant and Microbial Biology, University of California Berkeley, Berkeley, CA 94720, USA; US Department of Energy Joint Genome Institute, Lawrence Berkeley National Laboratory, Berkeley, CA 94720, USA; US Department of Energy Joint Genome Institute, Lawrence Berkeley National Laboratory, Berkeley, CA 94720, USA; US Department of Energy Joint Genome Institute, Lawrence Berkeley National Laboratory, Berkeley, CA 94720, USA; US Department of Energy Joint Genome Institute, Lawrence Berkeley National Laboratory, Berkeley, CA 94720, USA; US Department of Energy Joint Genome Institute, Lawrence Berkeley National Laboratory, Berkeley, CA 94720, USA; US Department of Energy Joint Genome Institute, Lawrence Berkeley National Laboratory, Berkeley, CA 94720, USA; US Department of Energy Joint Genome Institute, Lawrence Berkeley National Laboratory, Berkeley, CA 94720, USA

## Abstract

Algae are a diverse, polyphyletic group of photosynthetic eukaryotes spanning nearly all eukaryotic lineages of life and collectively responsible for ∼50% of photosynthesis on Earth. Sequenced algal genomes, critical to understanding their complex biology, are growing in number and require efficient tools for analysis. PhycoCosm (https://phycocosm.jgi.doe.gov) is an algal multi-omics portal, developed by the US Department of Energy Joint Genome Institute to support analysis and distribution of algal genome sequences and other ‘omics’ data. PhycoCosm provides integration of genome sequence and annotation for >100 algal genomes with available multi-omics data and interactive web-based tools to enable algal research in bioenergy and the environment, encouraging community engagement and data exchange, and fostering new sequencing projects that will further these research goals.

## INTRODUCTION

The Joint Genome Institute (JGI) is the Genomics User Facility funded by the US Department of Energy (DOE) to enable DOE mission relevant research in labs around the world. JGI provides access, at no cost, to high-throughput genomics and functional genomics capabilities including DNA and RNA sequencing, DNA synthesis, metabolomics, and data analysis through the JGI Community Science Program (CSP: https://jgi.doe.gov/user-programs/program-info/how-to-propose-a-csp-project/).

Algae are important players in carbon cycling, models for photosynthesis and sources for bioenergy and natural products. Algae provide 50% of global primary production as phytoplankton, coral symbionts, kelp forests and lichen symbionts (1). Algae have also driven eukaryotic evolution, by acquiring photosynthetic capacity multiple times through serial endosymbiotic events across the eukaryotic tree of life, and by giving rise to land plants (2). The first algal genome, that of the diatom *Thalassiosira pseudonana*, was sequenced by JGI (3), followed by several first-of-its-clade algal genomes, including the model green alga *Chlamydomonas reinhardtii* (4), the coccolithophore *Emiliania huxleyi* (5), and the nucleomorph-bearing cryptomonad *Guillardia theta* (6). Recently, with the development of new sequencing platforms and analytical tools, the JGI began a new strategic focus on exploring algal biology, diversity and evolution. We aim to accomplish this by scaling up algal genome sequencing and offering additional functional genomics and multi-omics capabilities.

Sequenced algal genomes with transcriptomes and other data are integrated into the JGI Algal multi-omics resource PhycoCosm (https://phycocosm.jgi.doe.gov), which currently includes over 100 algal genomes across the eukaryotic tree of life and can be explored interactively using the PhycoCosm web-based analytical tools. Algal data can be explored in the context of individual genomes, comparative genomics, and community annotation. New data and tools are constantly being added to the portal to contribute to the largest interactive collection of algal sequences. The JGI CSP calls for proposals enable efficient development of new resources and new collaborations.

## 100+ ALGAL GENOMES IN PHYCOCOSM, SPANNING THE EUKARYOTIC TREE OF LIFE

**Figure 1.**
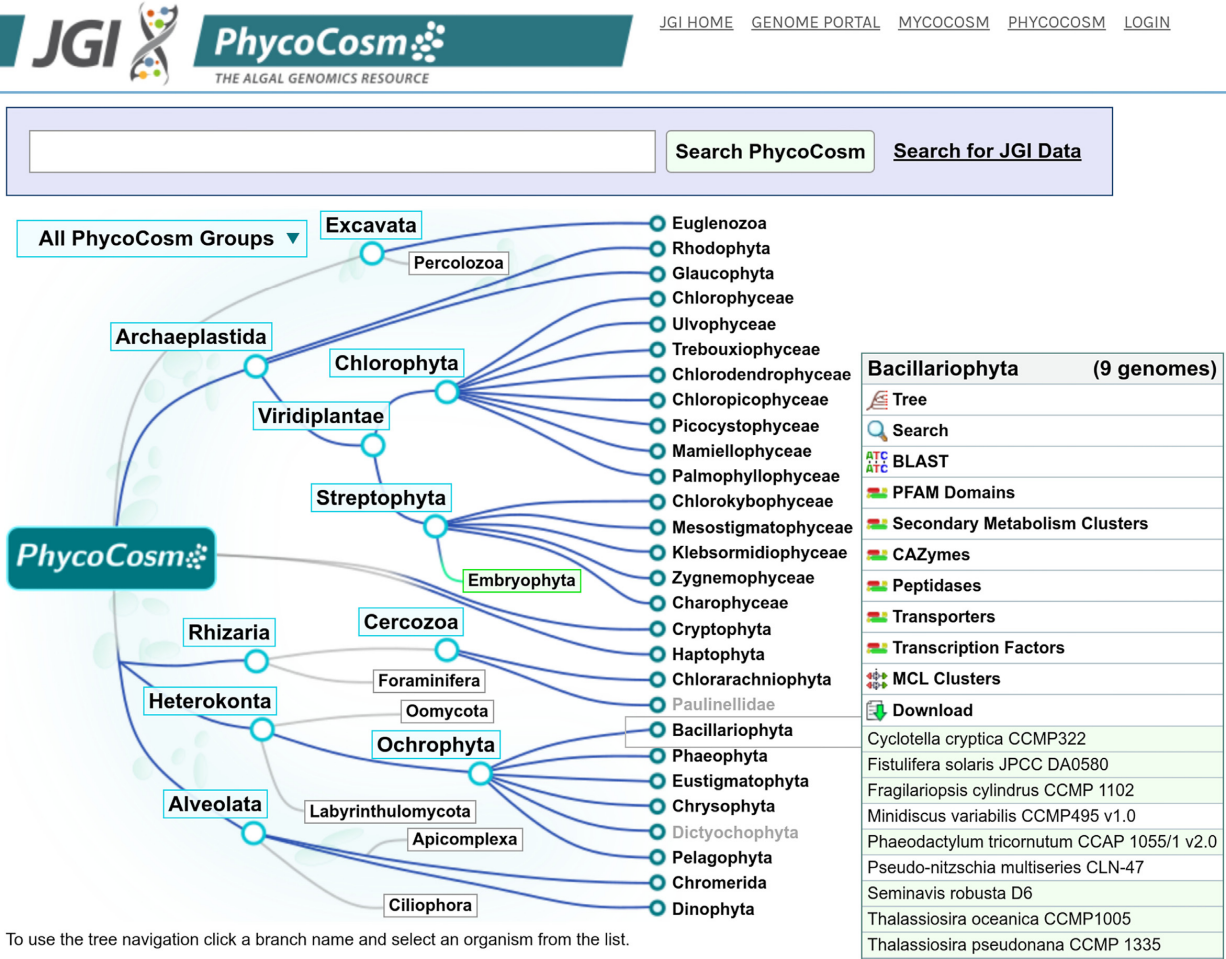
PhycoCosm Navigator with the Bacillariophyta leaf node clicked to show the drop down menu with a list of sequenced genomes, publication status (green if genome has been published), annotation and analysis tools, and the menu header ‘Bacillariophyta’ linking to the corresponding PhyloGroup.

The PhycoCosm *Navigator* (Figure 1) displays the major eukaryotic clades containing algal and other species with sequenced genomes and defines the scope for comparative analysis. The Navigator's root node displays all genomes at once, facilitating explorations of phylogenies, gene families, and functional annotations. The same analyses are available for smaller groups, and individual genomes, moving from the root to the leaves representing different algal groups. On the right side of the Navigator each leaf node shows available genomes to explore with a set of tools listed above it. The Search function allows users to type an organism name or part of it and jump directly to a specific genome without browsing.

PhycoCosm hosts both JGI-sequenced and annotated genomes and those imported to our database from external sources, including a recent addition of the genomes from Streptophyta and Chlorophyta. Other external genomes have increased the phylogenetic depth of existing nodes, such as Bacillariophyta, Eustigmatophyta, and Haptophyta, among others. (Figure 1). PhycoCosm is linked to plant genomes in the JGI Phytozome via the Embryophyta node (7). The supplemental nodes (Embryophyta, Percolozoa, Foraminifera, Oomycota, Labyrinthulomycota, Apicomplexa, Ciliophora) provide important comparative genomics context for the study of evolution of photosynthesis and adaptation to different habitats and ecological lifestyles.

## GENOME BROWSING AND MULTI-OMICS DATA DISPLAY

Multi-omics research is expanding rapidly beyond genome sequencing and includes a multitude of different techniques. Each algal genome in PhycoCosm has its own *genome-centric view* where genomics and other ‘omics’ data can be explored in depth, in a single-genome context. A menu at the top provides links to general information about the organism/genome statistics (home and info pages) and download section, BLAST (8) and keyword search functions, genome browser, functional annotations, and comparative tools discussed further down.

**Figure 2.**
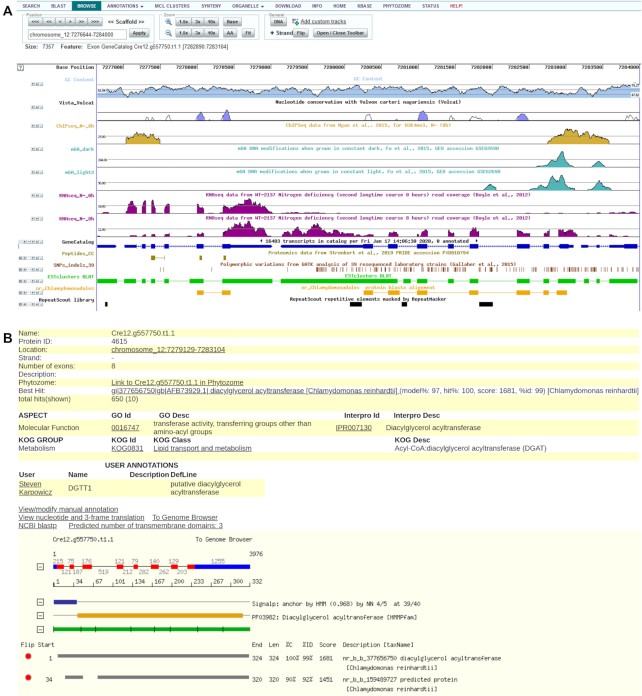
(**A**) The PhycoCosm genome browser view of the *Chlamydomonas reinhardtii DGTT1* locus shows an acyltransferase involved in triacylglycerol accumulation and induced by nitrogen (N) starvation. Visible tracks are a sample of those possible. The GeneCatalog track shows the *DGTT1* gene (right side) and a downstream cell cycle gene model (left side). The two RNAseq tracks 0 and 8 h after N starvation show that *DGTT1* is induced while the cell cycle gene is not (9). The ChIPseq and 6mA tracks show H3K4me3 histone and N^6^-deoxymethyladenine (6mA) DNA modifications, respectively, at the 5′ end of *DGTT1*. The light and dark 6mA tracks suggest light regulation (10). The SNPs track displays polymorphisms found by resequencing 39 strains (11). The Peptides track from a cell cycle proteomics study supports the cell cycle gene model (12). (**B**) The protein page for the *C. reinhardtii DGTT1* locus shows an expressed multi-exonic gene (CDS in red, UTR regions in blue, and translation in green) that encodes a membrane-anchored acyltransferase, as determined by automated annotation (secretion signal prediction in blue, predicted domain in orange, and BLAST alignments in gray) and manual curation (user annotation). The protein page links back to the genome browser (A) and a corresponding Phytozome page.

The PhycoCosm Browse tab (Figure 2A) provides a centralized location to display a variety of multi-omics data, mapped to the genome assembly, for deeper exploration on a per-locus basis. When coupled with gene model tracks, genome conservation, and other genomic features, these data provide powerful insights into gene regulation as well as evidence to support (or invalidate) predicted gene models. The genome browser (Figure 2A) is based on a version of the UCSC Genome Browser (13) with a configurable selection of tracks to show gene predictions and different lines of evidence in support of those predictions, including alignments with RNA, proteins, and other genomes. Additional tracks can display other features identified by other post-genomic experiments, such as resequencing, proteomics, and epigenomics. The navigation menu at the top allows users to move between scaffolds and positions, zooming, and retrieval of the underlying sequence. The toolbar controls track visibility and order, and provides options to save the current view and to create publication-quality images. Each track can be expanded to provide more details about the features contained within. Clicking a gene model connects to a cognate protein page (Figure 2B). Protein pages show model coordinates, structure and functional annotations such as protein domains and signal peptide predictions, manual curations, and links to external resources. Each protein page also links to a cognate annotation page, where registered users can manually curate gene models by adding deflines, gene names, experimental evidence, and literature citations. User annotations are publicly displayed on protein pages (Figure 2B), credited to their author, and become searchable. The genomes sequenced by JGI and deposited in GenBank are linked back to the PhycoCosm protein pages via the ‘JGIDB’ field (http://www.ncbi.nlm.nih.gov/genbank/collab/db_xref/); manual curations applied before submission are deposited in NCBI as well.

**Figure 3.**
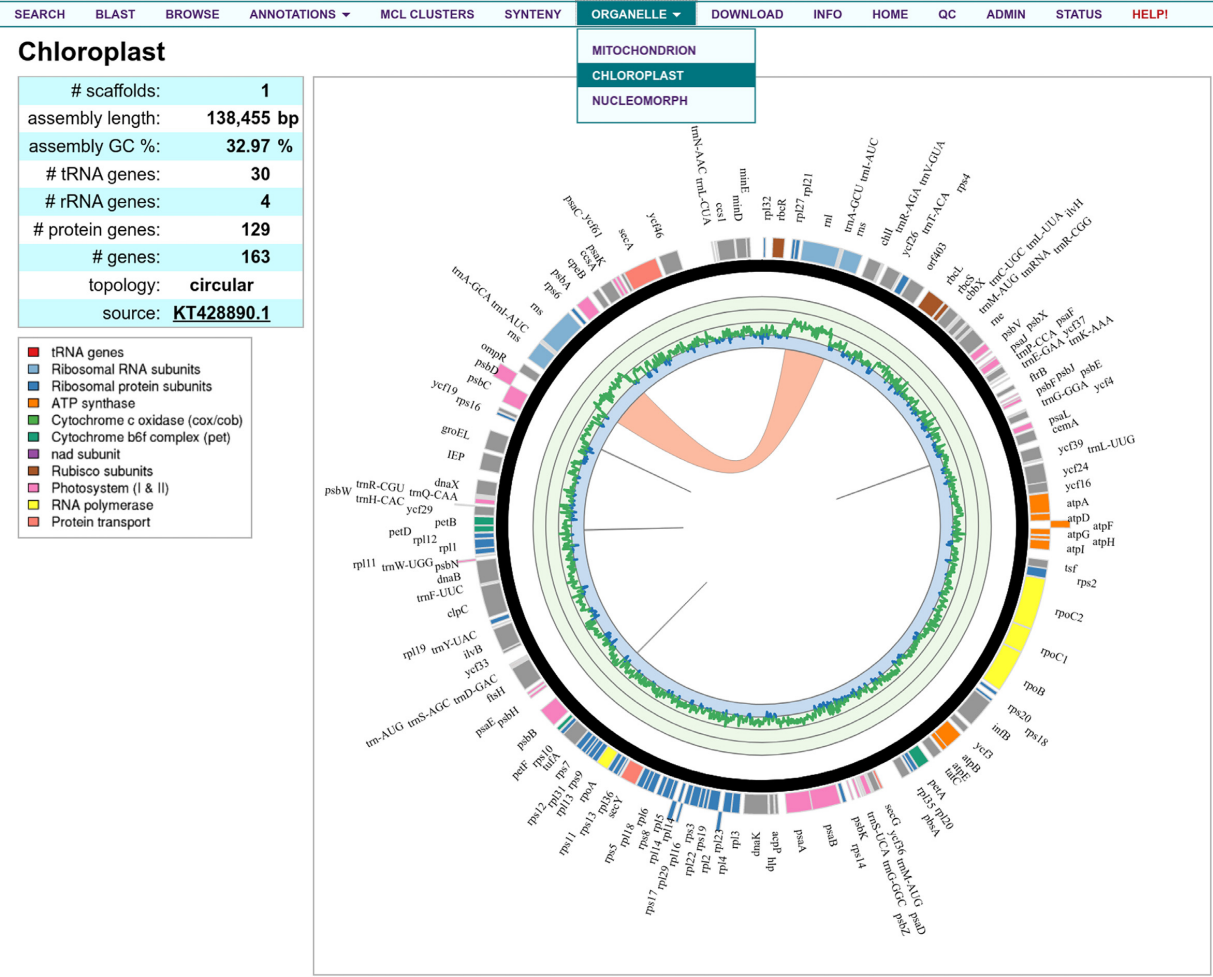
Circular representation of the *Guillardia theta* plastid genome. Gene models are along the outside of the ring and colored according to the legend. The inner line graph represents average %GC across the genome, ranging (inner to outer) from 0 to 100%. Using the dropdown menu on the navigation toolbar, users are also able to view the mitochondrial and nucleomorph genomes (6). Genomes are plotted using Circos (16).

Multi-omics analyses allow reconstruction of gene networks, metabolic modeling, and cross-platform integration. When available, a genome-centric view has a KBase tab (Figure 2A) to link to modeling tools in the DOE Systems Biology Knowledge Base, KBase (14), where users can access publicly available metabolic models (e.g. 15), and run analyses such as flux balance analysis (FBA) to predict the flow of metabolites through a metabolic network. For shared Archaeplastida, the genome-centric view's Phytozome tab (Figure 2A) links to the corresponding Phytozome (7) tools for analysis in a land-plant context; similarly, protein pages are crosslinked (Figure 2B). When available, a genome-centric view's Organelle tab displays circular plots of annotated mitochondrial, plastid, and when present, nucleomorph genomes (Figure 3).

## COMPARATIVE GENOMICS

**Figure 4.**
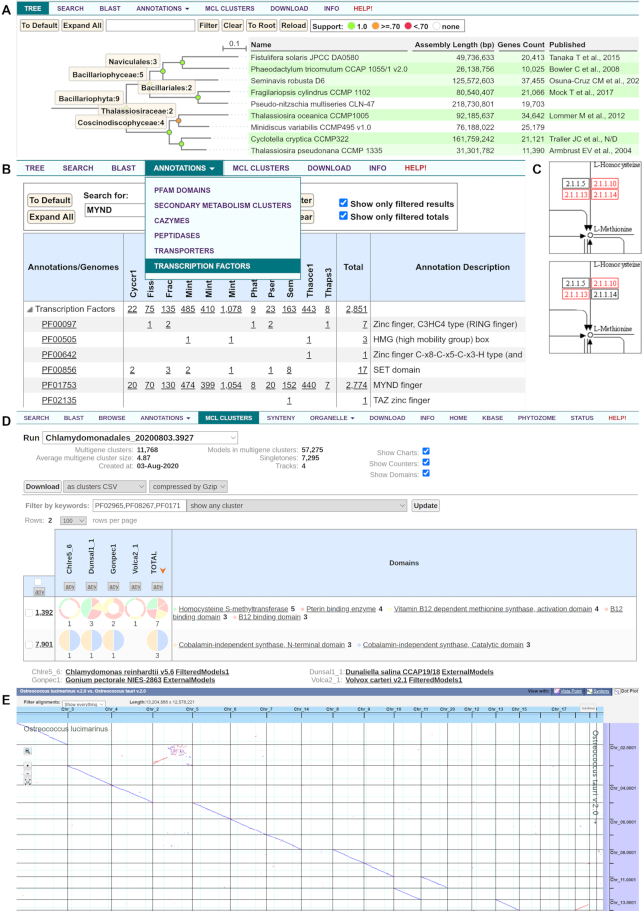
The PhycoCosm comparative tools. (**A**) The Tree tab for the Bacillariophyta PhyloGroup illustrates the classical division of diatoms between Coscinodiscophyceae and Bacillariophyceae (17). (**B**) The Annotation tab's Transcription Factors feature for the Bacillariophyta PhyloGroup shows that the abundance of transcription factors with MYND finger domains is highly variable among diatoms (18). (**C**) KEGG maps shows annotation of EC 2.1.1.14, cobalamin-independent methionine synthase, for *C. reinhardtii* (on top, box colored red), but not for *Volvox carteri* (on bottom, box colored black), consistent with the experimental observation of vitamin B12 auxotrophy in *V. carteri* but not in *C. reinhardtii* (19,20). (**D**) The MCL Clusters tab confirms that the cobalamin-dependent methionine synthase gene family is shared by both *C. reinhardtii* and *V. carteri*, but *V. carteri* does not possess the cobalamin-independent methionine synthase, thus complementing the KEGG pathway analysis feature (C). (**E**) The Synteny tab's dotplot visualizes high synteny between two *Ostreococcus* genomes with some genome reshuffling between chromosomes 2 of both species and the higher numbered chromosomes (21).

In addition to the tools for in-depth exploration of individual genomes, PhycoCosm assembles genomes into groups by phylogeny (*PhyloGroups*) and ecology (*EcoGroups*) for comparative genomics analyses (Figure 4). PhyloGroups facilitate comparative analyses of species with a shared evolutionary history. The Algae EcoGroup contains over 100 photosynthetic eukaryotes, with the exception of land plants, while additional EcoGroups include subdivisions by similar lifestyle (e.g., seaweeds) or environment (e.g. Arctic algae), and even cross-kingdom associations (e.g. lichens formed between algal and fungal symbionts). All groups, including non-photosynthetic taxa important for comparative genomics, are available from the drop-down list of the PhycoCosm Navigator. PhyloGroups have a Tree tab that provides a searchable and interactive phylogenetic tree for exploring the evolutionary relationships between the members of the group (Figure 4A). Each species tree is constructed from whole-genome protein alignments using maximum-likelihood (8,22). Individual nodes and leaves may be collapsed and expanded, and selected for statistical details and moving down the taxonomic hierarchy to other PhyloGroups, or to individual genome views.

Both genome-centric and group views have an Annotations tab for accessing multiple categories of functional annotations of predicted genes in multiple genomes including *peptidases* based on MEROPS (23), *transporters* based on TCDB (24), *Transcription Factors* based on literature and web resources such as TAPscan (25–27) (Figure 4B), and others. Each category is offered as a table of functional annotations (e.g. MEROPS names, TCDB families, transcription factor domains, etc) with protein counts for each annotation by genome, allowing direct comparison of functional assignments. Genome-centric views also include GO (28), KOG (29) and KEGG (30) (Figure 4C) annotations.

The MCL Clusters tab links to pre-calculated homology-based protein clusters for gene family analysis (Figure 4D). Clusters are generated by converting all-vs-all protein alignments between species into a distance matrix and building protein clusters using MCL (8,31). Each cluster is exhibited as a row in a table with columns of genomes, displaying both gene counts and distributions of predicted protein domains. The annotation and gene family tables may be browsed, searched, filtered, and sorted, allowing identification of gene family expansions, contractions, and absences in or from individual genomes. For more detail, individual cluster page links may be followed, listing the genes that comprise the cluster and graphically visualizing the exon-intron structures of the member genes, their domain structures, and their relative chromosomal positions in a synteny view. In addition, the Synteny tab of a genome-centric view displays VISTA whole-genome alignments (32) (Figure 4E).

## ALGAL GENOME ANNOTATION CHALLENGES AND FUTURE DEVELOPMENTS

Algal genomes can be annotated using eukaryotic annotation pipelines (e.g. 33) with significant tuning since genomic properties of algae are highly variable. For example, the *E. huxleyi* genome has ∼55% of non-standard GC donor splice sites, challenging most *ab-initio* and homology-based gene predictors (5). Euglenophyta conduct wide-spread trans-splicing events (34). Dinoflagellate genomes are very large and complex, posing great challenges for sequencing. Many algal clades, amongst the Rhizaria, Heterokonta, Alveolata and Euglenozoa, are poorly represented by sequenced genomes or well-characterized genes, making ∼700 marine microbial eukaryotic transcriptomes (MMETSP) (35,36) critical for discovery of unique algal features and annotation of algal genomes.

Plastids are the defining feature of algae. Historically, JGI comparative genomics resources have focused on nuclear genomes, but we have started to add tools for organelle genome exploration. For annotation of chloroplast genomes, we developed a new approach combining *ab initio*, homology and HMM-based gene prediction methods, based on manually curated multiple sequence alignments for all the conserved chloroplast gene families. The plastid proteins encoded in the nuclear genome and post-translationally targeted to the plastid using N-terminal targeting peptides can be predicted with a combination of cellular localization predictors, both general (e.g. WoLF PSORT (37), TargetP (38), DeepLoc (39)) and lineage-specific (e.g. PredAlgo (40) for green algae, HECTAR (41) for heterokonts and ASAFind (42) for diatoms). These plastid genes in nuclear genomes are the result of horizontal gene transfer (HGT) after primary or secondary endosymbiosis (43). HGT may be even more wide-spread, beyond plastid genes, in Chlorarachniophyta (44), Cryptophyta, Rhizaria, Alveolata, Heterokonta and Haptophyta (45), and is to be distinguished from bacterial contamination. To identify genes that result from HGT, we developed a method based on incongruences between gene and species trees (46).

Integration of 100+ annotated algal genomes sequenced at JGI and elsewhere into a single comparative genomics resource, PhycoCosm, is a first step toward comprehensive analysis of algal genome data. Comparing these genomes from different sources annotated with different strategies and tools enables efficient quality checks and development of better methods for algal annotation. PhycoCosm offers an interactive platform for data distribution, visualization, and analysis, and enables annotation of different genomes to the same standards regardless of where they were sequenced and assembled. Filtering out artifacts from original gene sets and use of the same reference databases for functional annotation will bring different gene sets to similar standards, and make them more comparable and less dependent on annotation strategy. A dramatic scale-up in algal genomics in the next few years will require better visualization and more efficient analysis tools. PhycoCosm offers a one-stop shopping point for both multi-omics data and multidimensional genomics analyses.

## DATA AVAILABILITY

PhycoCosm data is available at https://phycocosm.jgi.doe.gov.
